# Effectiveness and Safety of Rituximab in Recalcitrant Pemphigoid Diseases

**DOI:** 10.3389/fimmu.2018.00248

**Published:** 2018-02-19

**Authors:** Aniek Lamberts, H. Ilona Euverman, Jorrit B. Terra, Marcel F. Jonkman, Barbara Horváth

**Affiliations:** ^1^Center for Blistering Diseases, Department of Dermatology, University Medical Center Groningen, University of Groningen, Groningen, Netherlands

**Keywords:** pemphigoid diseases, autoimmune bullous diseases, rituximab, IgA, mucous membrane pemphigoid, linear IgA disease, epidermolysis bullosa acquisita, case series

## Abstract

**Introduction:**

Rituximab (RTX) is a monoclonal antibody targeting CD20, a transmembrane protein expressed on B cells, causing B cell depletion. RTX has shown great efficacy in studies of pemphigus vulgaris, but data of pemphigoid diseases are limited.

**Objective:**

To assess the effectiveness and safety of RTX in pemphigoid diseases.

**Methods:**

The medical records of 28 patients with pemphigoid diseases that were treated with RTX were reviewed retrospectively. Early and late endpoints, defined according to international consensus, were disease control (DC), partial remission (PR), complete remission (CR), and relapses. Safety was measured by reported adverse events.

**Results:**

Patients with bullous pemphigoid (*n* = 8), mucous membrane pemphigoid (*n* = 14), epidermolysis bullosa acquisita (*n* = 5), and linear IgA disease (*n* = 1) were included. Treatment with 500 mg RTX (*n* = 6) or 1,000 mg RTX (*n* = 22) was administered on days 1 and 15. Eight patients received additional 500 mg RTX at months 6 and 12. Overall, DC was achieved in 67.9%, PR in 57.1%, and CR in 21.4% of the cases. During follow-up, 66.7% patients relapsed. Repeated treatment with RTX led to remission (PR or CR) in 85.7% of the retreated cases. No significant difference in response between pemphigoid subtypes was found. IgA-dominant cases (*n* = 5) achieved less DC (20 vs. 81.3%; *p* = 0.007), less PR (20 vs. 62.5%; *p* = 0.149), and less CR (0 vs. 18.8%; *p* = 0.549) compared to IgG-dominant cases (*n* = 16). Five severe adverse events and three deaths were reported. One death was possibly related to RTX and one death was disease related.

**Conclusion:**

RTX can be effective in recalcitrant IgG-dominant pemphigoid diseases, however not in those where IgA is dominant.

## Introduction

Pemphigoid diseases are a heterogeneous group of autoantibody-mediated subepidermal blistering diseases (1). IgG, IgA, or IgM autoantibodies target distinct antigens located in the basement membrane zone (BMZ) inducing different pemphigoid subtypes. Cutaneous pemphigoid is the subgroup of pemphigoid diseases that predominantly affect the skin (1, 2). Pemphigoid, be it non-bullous or bullous (BP), is the most prevalent disease within this subgroup and mainly presents at older age (3). Mucous membrane pemphigoid (MMP) opposes cutaneous pemphigoid in the spectrum of pemphigoid diseases, and is characterized by primary involvement of the mucosa (2, 4). Beside the classification based on body localization, pemphigoid diseases may be classified based on targeted auto-antigens, such as 180 kDa BP antigen (BP180) and the 230 kDa BP antigen (BP230) in pemphigoid, laminin-332 in anti-laminin-332 MMP, the p200 protein in anti-p200 pemphigoid (anti-laminin γ1 pemphigoid), and type VII collagen in epidermolysis bullosa acquisita (EBA) (2). Last, classification may be based on predominant class of autoantibodies IgG or IgA. Pemphigoid diseases with the exclusive IgA involvement are named linear IgA disease (LAD), regardless of the targeted antigen or clinical presentation (5). IgA-mediated pemphigoid diseases are difficult to treat if dapsone is contraindicated, and mostly show high resistance to usual immunosuppressants (6).

The 2014 European consensus guideline for the management of BP recommends transcutaneous systemic clobetasol therapy as initial treatment (7, 8). The alternative is oral systemic prednisolone therapy (0.5–1.0 mg/kg/day), which was associated with adverse events and higher mortality (8, 9). Recently, doxycycline was found to be non-inferior to and safer than prednisolone for short-term blister control (10), although the statistical margins were wide (11). As third-line rituximab (RTX) is recommended in cases in which conventional immunosuppressive drugs were not effective, were contraindicated, or showed unacceptable side effects (8, 12, 13).

Rituximab is a chimeric monoclonal antibody targeting CD20, a transmembrane protein expressed by all B cells in the pre-plasma cell lineage (14). Binding of RTX to CD20 leads to B cell depletion in the peripheral circulation by various mechanisms (15). RTX is registered for treatment of B cell lymphoma’s, rheumatoid arthritis (RA), and granulomatosis with polyangiitis (15, 16). Recently, RTX was shown to be effective as first-line therapy in pemphigus (17). However, the position of RTX on the therapeutically ladder of pemphigoid diseases is unknown. Data regarding the effectiveness and safety of RTX in pemphigoid diseases are limited and are mainly of retrospective nature (18–26). Furthermore, it is unclear which treatment regime is most beneficial and which factors might be predictive for treatment response (27, 28). Therefore, the aim of our study was to retrospectively analyze our daily practice experience with RTX in pemphigoid diseases by evaluating the effectiveness and safety, and to identify clinical or serological factors that might be associated with treatment response.

## Materials and Methods

All patients with pemphigoid diseases treated with RTX between 2010 and September 2017 at the Center for Blistering Diseases at the University Medical Center Groningen were included in the study. Pemphigoid diseases were diagnosed based on the following criteria: linear depositions of IgG, IgA, IgM, or C3c along the BMZ by direct immunofluorescence microscopy (DIF) and/or positive indirect immunofluorescence microscopy (IIF) on monkey esophagus (MO) or salt-split skin (SSS), in combination with clinical presentation, histopathological findings, or other immunoserological tests compatible with the diagnosis of a pemphigoid disease. Patients with a linear u-serrated immunodeposition pattern seen by DIF were diagnosed with EBA. Patients with exclusive involvement of IgA were diagnosed with LAD.

Patients charts were reviewed retrospectively by the first (Aniek Lamberts) and second (H. Ilona Euverman) authors. Response outcomes were defined according to international consensus and measured by the early endpoint disease control (DC), and the late endpoints partial remission (PR), complete remission (CR), and the number of relapses (29, 30). Safety was measured by reported adverse events. Discrepancies in the assessment by Aniek Lamberts and H. Ilona Euverman were resolved through discussion with the other authors (Barbara Horváth and Marcel F. Jonkman).

### Treatment Regimes

There were two treatment protocols administered. In the period of 2010–2012, patients were treated with RTX 500 mg at days 1 and 15 (low-dose RA protocol), since this dose was effective in pemphigus patients (Horvath et al. 2011) (31, 32). Additional 500 mg RTX at month 6 and/or 12 was only administered on indication (33). From 2012 the protocol was adjusted to 1,000 mg RTX at days 1 and 15 (high-dose RA protocol—published in 2011) (28). Since 2014, patients standardly received additional 500 mg RTX at months 6 and 12, and if indicated at month 18. Patients that relapsed within 1 year after the last RTX infusion received re-treatment with a single infusion of 500 mg RTX. Relapsed patients beyond 1 year after the last RTX infusion were retreated with a new cycle of 1,000 mg RTX at days 1 and 15.

### Statistical Analysis

The Kolmogorov–Smirnov test was used to test for normal distributions. Correlations between bivariate outcome measures were analyzed with Fisher’s exact test. Comparing means of non-normally distributed data was done with the Mann–Whitney *U* test. Statistical significance was defined by a *p*-value <0.05. Statistical analyses were performed in IBM SPSS statistics version 23.

## Results

### Patient Population

A total of 28 patients were included. The patient characteristics are listed in Table 1. The mean delay in diagnosis was 10.5 months in BP (range 1–19), 24.3 months in MMP (range 4–60), and 19.0 months in EBA patients (range 3–47). One MMP outlier with an exceptional long delay in diagnosis of 285 months was not taken into account. This patient showed severe laryngeal, oral, genital, and ocular (foster stage 4) involvement. The mean time between diagnosis and RTX treatment was longer for BP patients (64.3 months; range 1–272), EBA (29.1 months; range 0.5–84), and LAD patients (49.0 months) compared to MMP (13.8 months; range 2–63). Prior to RTX, all patients received one or more immunosuppressants (Table S1 in Supplementary Material) with suboptimal effect or with unacceptable side effects. Therefore, RTX was administered as last resort in several cases. Six patients received low-dose RTX (500 mg) and 22 patients high-dose RTX (1,000 mg), of which eight patients also received repeated RTX doses (500 mg) at months 6 and 12. In all patients RTX was added to pre-existing treatment with a local steroid and/or one or two systemic drugs (Table S1 in Supplementary Material).

**Table 1:** Demographics of pemphigoid patients treated with RTX.

Mean age at first cycle RTX	BP (*n* = 8)^a^	67.13 years	SD 9.4, range 53–78

	MMP (*n* = 14)	64.9 years	SD 12.3, range 45–84
	Ocular involvement (*n* = 7)^b^		
	Oral involvement (*n* = 11)		
	Laryngeal involvement (*n* = 4)		
	Genital involvement (*n* = 2)		

	EBA, all inflammatory subtype (*n* = 5)	54.0 years	SD 22.8, range 25–87
	LAD (*n* = 1)	48.0 years	–
	Total (*n* = 28)	63.0 years	SD 14.3, range 25–87

Dominant Ig in DIF and IIF on SSS	IgG dominant	16 patients	
	IgA dominant	5 patients	
	IgM dominant	1 patient	
	IgG/IgA equally dominant	6 patients	

Gender	Male	13 (46.4%)	
	Female	15 (53.6%)	

First cycle of 2 × 500 mg		6 patients	
	Additional cycle 2 × 1,000 mg	3 patients	
	Additional cycle 2 × 500 mg	1 patient	

First cycle of 2 × 1,000 mg		22 patients^c^	

	Additional cycle 2 × 1,000 mg	1 patients	
	Additional cycle 2 × 500 mg	1 patient	
Additional gifts of RTX	500 mg at M6 and/or M12	15 patients^d^	
	500 mg at M6 and M12	8 patients	

Mean total follow-up time (first RTX cycle till last contact)		30.3 months	SD 23.0, range 2–79

*RTX, rituximab; BP, bullous pemphigoid; MMP, mucous membrane pemphigoid; EBA, epidermolysis bullosa acquisita; LAD, linear IgA disease; Ig, immunoglobulin; DIF, direct immunofluorescence microscopy; IIF, indirect immunofluorescence microscopy; SSS, salt-split skin; M6, month 6; M12, month 12*.

*^a^All patients with pemphigoid presented with the BP*.

*^b^Two patients had exclusive ocular involvement, also known as pure ocular MMP*.

*^c^One patient only received 1 × 1,000 mg due to the development of pneumocystis pneumonia*.

*^d^Five patients only received 500 mg RTX at M6, two patients only received 500 mg RTX at M12*.

### Effectiveness of First Course of RTX

**Figure 1:**
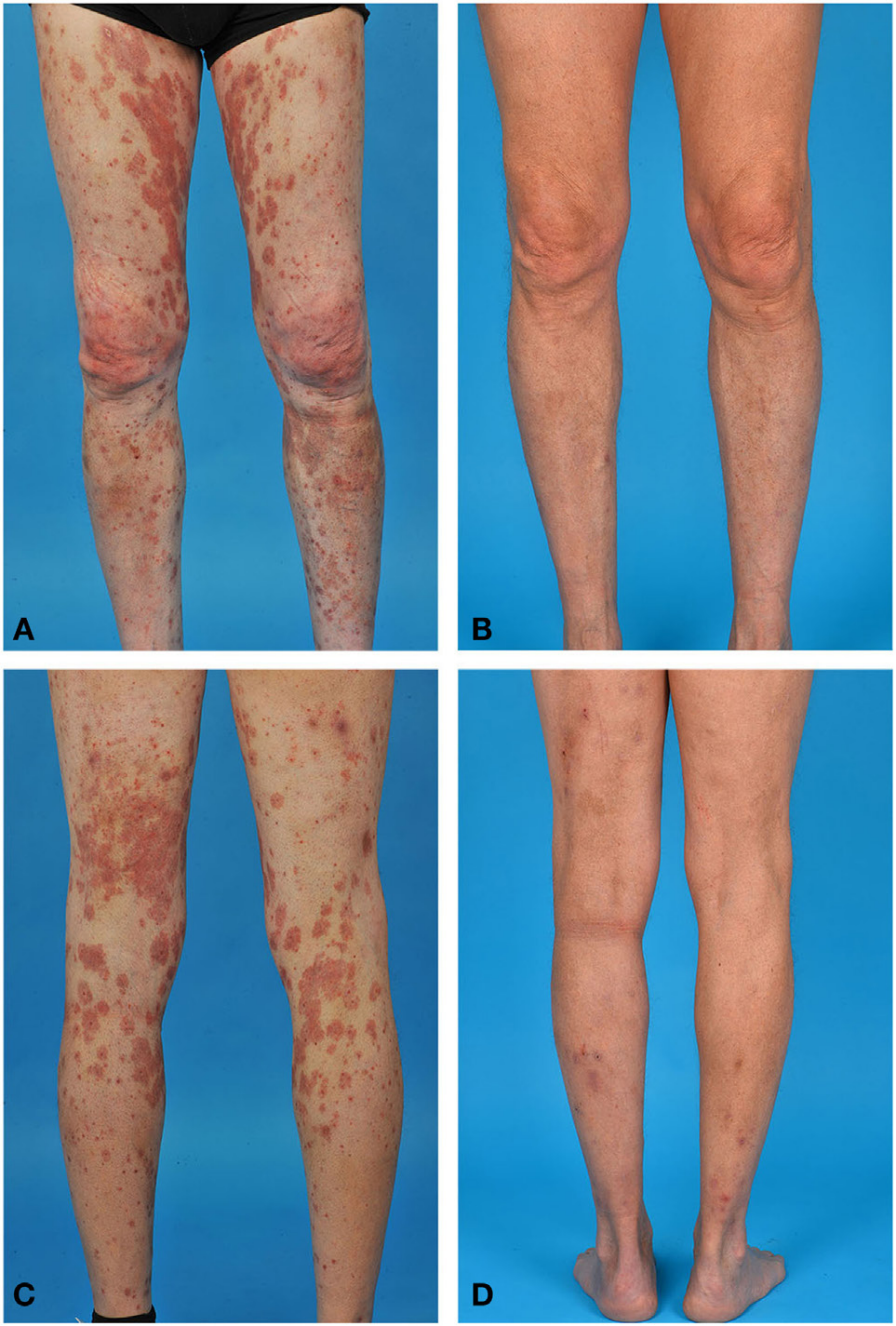
Bullous pemphigoid in a 69-year-old male. **(A,C)** Erythematous plaques and papules on both legs before rituximab (RTX) treatment. **(B,D)** Remission with minimal therapy after RTX treatment.

**Figure 2:**
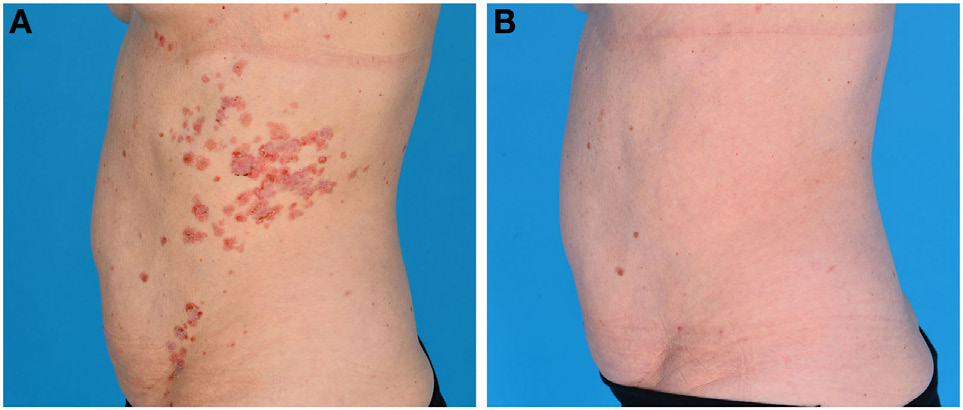
Epidermolysis bullosa acquisita (EBA) in a 59-year-old female. **(A)** Nummular erythematous plaques, papules and circinate configurated crustae, vesicles, and bullae on the trunk, before rituximab (RTX) treatment. **(B)** Remission off therapy after RTX treatment.

**Figure 3:**
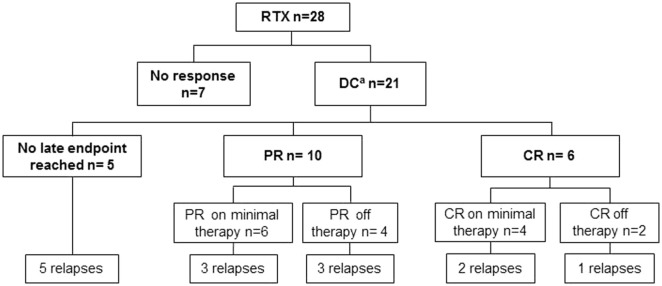
Flowchart of the effectiveness of RTX in pemphigoid patients, showing the highest endpoint reached after the first RTX cycle. RTX, rituximab; DC, disease control; PR, partial remission; CR, complete remission. ^a^Two patients already achieved DC before RTX was administered.

**Figure 4:**
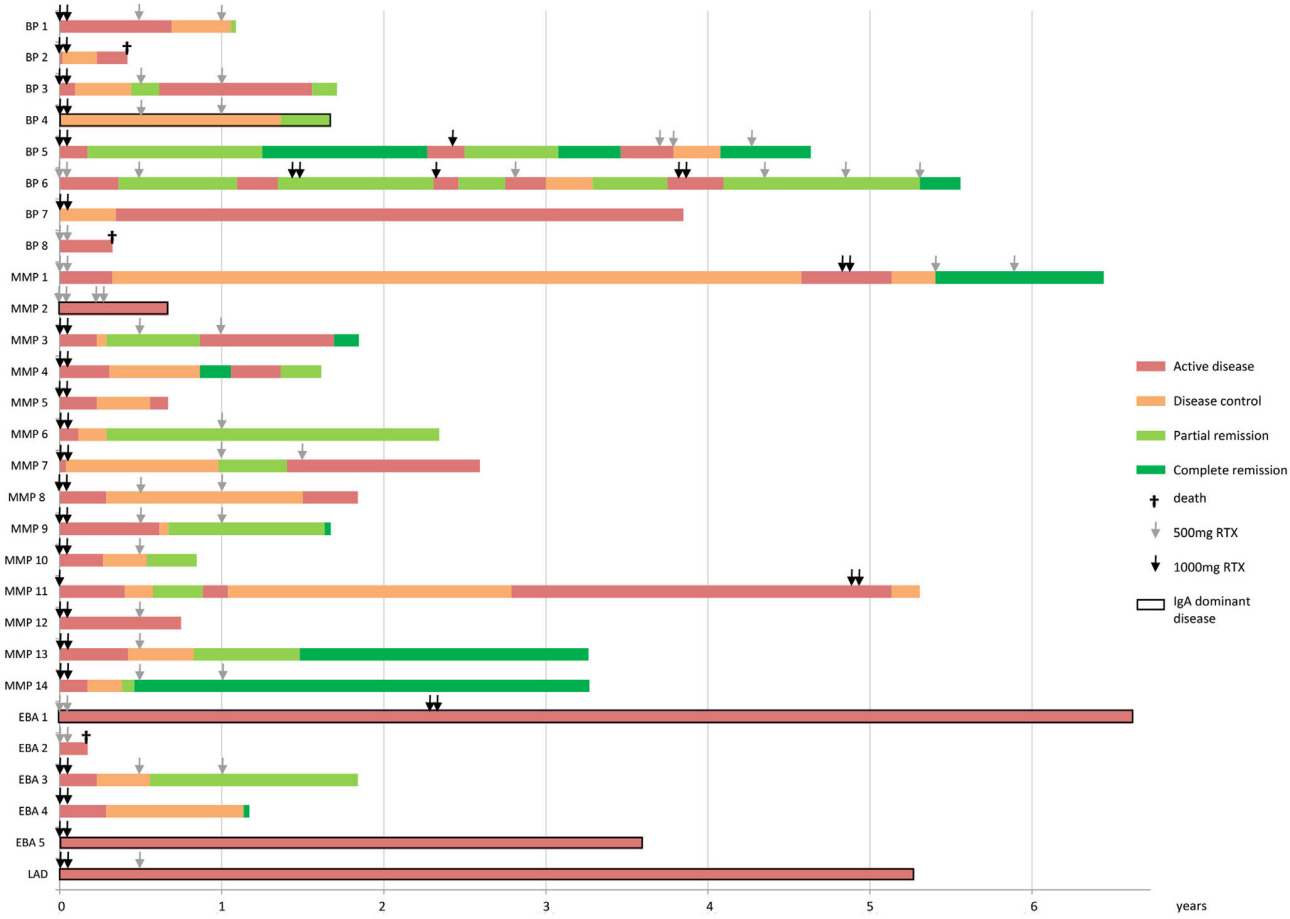
Bar chart showing the achieved endpoints and repeated treatment of RTX of each individual pemphigoid patient. Bars represent patients until the end of follow-up. The pemphigoid subtypes are indicated on the *y*-axis and the *x*-axis displays time in years. RTX, rituximab; BP, bullous pemphigoid; MMP, mucous membrane pemphigoid; EBA, epidermolysis bullosa acquisita; LAD, linear IgA disease.

DC was achieved in 19 of 28 patients (67.9%) at a mean time of 14.5 weeks (range 1–36; SD 9.1). Remission (partial or complete) was achieved by 57.1% (*n* = 16) of the treatment resistant pemphigoid cases (Figures 1 and 2). PR was achieved by 16 patients (57.1%) at a mean time of 34.2 weeks (range 9–71; SD 18.1). Six of 28 patients (21.4%) also achieved CR at a mean time of 59.2 weeks (range 24–85; SD 22.1). Figures 3 and 4 display a flowchart and bar chart of the achieved early and late endpoints during follow-up. A complete overview of the outcome measurements of all included patients can be found in Table S1 in Supplementary Material.

We analyzed whether early administration of RTX was more beneficial. We compared the mean time between onset of symptoms and RTX treatment of patients with PR or CR (52.2 months; *n* = 16) and patients without PR or CR (64.9 months; *n* = 12). No significant difference was found [Mann–Whitney test (*p* = 0.642)].

### Comparison of Treatment Regimes

Significantly more patients achieved DC with 1,000 mg RTX at days 1 and 15 (85.0%) compared to 500 mg RTX (33.3%; *p* = 0.028). Furthermore, patients more often achieved PR (63.6 vs. 16.7%; *p* = 0.057) and CR (27.3 vs. 0%; *p* = 0.289). Relapses were seen in both two cases receiving 500 mg RTX and 12 out of 19 cases (63.2%) with 1,000 mg RTX (*p* = 0.533).

Patients receiving repeated RTX infusions (*n* = 8) achieved DC (100 vs. 63.2%; *p* = 0.134) and PR (87.5 vs. 45.0%; *p* = 0.088) more frequently than patients without additional RTX infusions. A similar number of patients achieved CR (25.0 vs. 20%; *p* = 1.000). The relapse rate in the group with the additional gifts was lower (50.0 vs. 76.9%; *p* = 0.346), and the mean time until relapse was longer [81.3 weeks (range 32–170; SD 62.3) vs. 69.0 weeks (range 12–238; SD 66.6); *p* = 0.572].

### Response in the Different Pemphigoid Subtypes

Mucous membrane pemphigoid patients showed the most benefit of RTX with DC in 85.7%, PR in 64.3%, and CR in 28.6% patients. During follow-up, 75% of the MMP patients relapsed. BP patients achieved DC in 83.3%, PR in 62.5%, and CR in 12.5% with a relapse rate of 71.4%. In EBA patients, DC was found in 40%, PR in 40%, and CR in 20% without relapse. The patient with LAD was unresponsive to RTX treatment. No significant difference was found in the effectiveness of RTX between the different pemphigoid subtypes by Fisher’s exact test.

### Immunological Findings

The dominant immunoglobulin class prior to RTX treatment assessed by staining intensity in DIF and IIF on SSS was IgG in the majority of the cases (57.1%; *n* = 16), IgA in 17.9% (*n* = 5), and IgM in 3.6% (*n* = 1). Equal intensity of IgA and IgG staining was observed in 21.4% (*n* = 6) of the cases. IgA-dominant pemphigoid cases (*n* = 5) showed significantly less DC (20 vs. 81.3%; *p* = 0.007) compared to IgG-dominant cases (*n* = 16). The proportion of patients achieving PR (20 vs. 62.5%; *p* = 0.149) and CR (0 vs. 18.8%; *p* = 0.549) did not differ significantly; however, it showed a clear trend of ineffectiveness of RTX in IgA-dominant cases. Post treatment analysis was not performed.

Deposition of C3c along the BMZ was seen in 67.9% (*n* = 19) of DIF biopsies. No differences in effectiveness of RTX were found between patients with or without complement depositions.

### Relapses

Fourteen of 21 patients (66.7%) relapsed after a mean time of 72.5 weeks (range 12–238; SD 63.2). Four of 14 relapsed patients showed B cell repopulation; in one case repopulation preceded relapse and in three cases repopulation was objectified after the relapse. Five of 14 relapsed patients showed maintained B cell depletion and in five patients B cells were not followed up. Seven of 14 relapsed patients were retreated with RTX, which led to PR or CR in six out of seven patients (85.7%).

### Safety

Table 2 provides an overview of reported adverse events and deaths after RTX treatment. One patient died 4 months after RTX infusion due to sepsis which led to multi-organ failure. This death was interpreted as possibly related to RTX, since neutropenia (possibly RTX induced late onset neutropenia) might have contributed to a higher infection risk. All cases that reported grade 3 or grade 4 adverse events fully recovered. Five infusion reactions were observed in three patients during RTX administration: dyspnea with chest pain, tired feeling of the legs, and dizziness plus a burning sensation in the groins. All infusions could be successfully continued at lower infusion rate. One patient was accidently shortly infused with RTX subcutaneously, causing temporary pain and swelling of the arm.

**Table 2:** Adverse events and deaths reported in pemphigoid patients treated with rituximab (RTX).

		GRADE^a^	Concomitant immunosuppressive drugs
**Reported adverse events**			

Patient 1	Erysipelas right arm	3	Prednisolone 30 mg/day
	Herpes simplex labialis (confirmed HSV-1)	2	Prednisolone 30 mg/day

Patient 2,3	Upper respiratory infection probably viral (not confirmed)	1	Patient 2: prednisolone 10 mg/day
			Patient 3: none

Patient 4	PCP twice (no prophylaxis) - after first gift of 1,000 mg RTX - after second cycle of 2 × 1,000 mg RTX	4	Prednisolone 60 mg/day + cyclophosphamide 150 mg/day
		4	Prednisolone 20 mg/day

Patient 5	Urticaria e.c.i., self-limiting	1	Prednisolone 15 mg/day

Patient 6	Flare-up of concomitant psoriasis	2	Prednisolone 10 mg/day + dapsone 100 mg/day

Patient 7	Polyarthritis and fever, possibly caused by serum sickness (not confirmed)	3	Prednisolone 7.5 mg/day

Patient 8	Diarrhea and loss of consciousness, followed by hospitalization	3	Prednisolone 40 mg/day

Patient 9	Generalized pain e.c.i., self-limiting	2	Prednisolone 35 mg/day
	Urinary tract infection (female)	2	Prednisolone 35 mg/day

Patient 10	Upper respiratory infection probably viral (not confirmed)	2	Prednisolone 5 mg/day + cyclophosphamide 50 mg/day
	Urinary tract infection (male)	2	Prednisolone 5 mg/day + cyclophosphamide 50 mg/day

Patient 11	Myalgia e.c.i., self-limiting	1	Prednisolone 5 mg/day

**Deaths that occurred after RTX administration**			

Male, 78 years old, BP			Cognitive and physical decline. Exact cause of death unknown

Female, 73 years old, BP			Sepsis due to neglected urinary tract infection and neutropenia/leukopenia (possibly late onset neutropenia due to RTX), multi-organ failure eventually led to death

Female, 87 years old, EBA			Active disease with severe mucosal involvement, weight loss and physical decline, exact cause of death unknown (possibly disease related)

*RTX, rituximab; HSV-1, Herpes Simplex Virus type 1; PCP, pneumocystis pneumonia; e.c.i., e causa ingnota (of unknown cause); BP, bullous pemphigoid; MMP, mucous membrane pemphigoid; EBA, epidermolysis bullosa acquisita; LAD, linear IgA disease*.

*^a^Adverse events were graded according to the Common Terminology Criteria for Adverse Events v4.0 (CTCAE) (34)*.

### B Cell Depletion

B cells were undetectable in the peripheral blood within 2 weeks in all patients after a single RTX infusion. In 13 patients, B cell levels remained undetectable during a mean follow-up time of 77.5 weeks (range 24–269; SD 64.6). All 13 patients received repeated RTX infusions at months 6, 12, or both. Repopulation of B cells was seen in six patients after a mean time of 95.2 weeks (range 36–250; sd 82.4). B cell levels were not followed up in nine patients. B cell levels showed no clear relation with response to RTX. All data on B cell levels should be interpreted with caution, since B cells were not measured at standard time points in our study population.

## Discussion

Our study showed partial or CR with RTX in 57.1% of the cases with a pemphigoid disease, that previously failed on a variety of immunosuppressants. RTX was most beneficial in refractory MMP and BP patients with partial or CR in 64.3 and 62.5% of the cases. Interestingly, IgA-dominant pemphigoid diseases responded poorly on RTX.

Only two other studies described RTX treatment of MMP patients (*n* = 14; 11 cases with isolated ocular involvement) according to the RA protocol and showed PR or CR in all cases (19, 20). Both studies reported high relapse rates (83.3 and 100%); however, repeated treatment led to remission in all cases. These findings are in accordance with our observed relapse rate (66.7%) and remission rate after repeated RTX treatment (85.7%).

Previous studies on RTX therapy in MMP and BP reported either mixed responses with serious infectious adverse events and death (24–26), or high remission rates with limited non-serious adverse events (18–23). Studies on RTX in combination with immunoadsorption (protein A) or human intravenous immunoglobulin found high response rates in ocular MMP, resistant EBA, and recalcitrant BP (35–38). Interestingly, our study showed lower remission rates compared to most reports in the literature; DC in 100% (21), PR and/or CR in 66% (24), 86% (25), 88% (26), 92% (18), and 100% (23). These differences in the results can be explained by the clinical heterogeneity of the previous studies, caused by using different RTX regimes (21, 22, 24–26), by assessing different populations (multiple pemphigoid subtypes in a tertiary referral center in our study, MMP patients in most studies) (19–21, 26, 36), or by using different definitions for the outcome measurements (19, 22, 23, 39). Studies prior to the pemphigoid consensus of 2012 either used definitions of the pemphigus consensus of 2008, in which minimal adjuvant therapy was less well defined, or other definitions for treatment response (19, 22, 23, 39).

An important result is the observation of significantly more DC (*p* = 0.028), and more PR (*p* = 0.057) and CR (*p* = 0.289) in patients treated with a high dosage regime compared to a low dosage regime. Moreover, we noticed a beneficial effect of repeated RTX infusions with less relapses.

A major finding of our study is that four out of five (80%) IgA-dominant pemphigoid diseases were completely unresponsive to RTX treatment. Previously, He et al. demonstrated persistent IgA-secreting plasma cells in a MMP patient not responding to RTX treatment (40). They speculated that plasma cells could be derived from a tissue resident memory B cell population that is resistant to anti-CD20 therapy. Mei et al. described the continuous presence of IgA + plasma cells in the peripheral circulation and the gastrointestinal mucosa of RA patients during successful B cell depletion by RTX (41). Further characterization of the circulating plasma cells revealed a mucosal phenotype, indicating that their precursor B cells are mucosal resident, and not depleted by RTX. All these findings could explain the unresponsiveness in our IgA-dominant cases.

Fourteen adverse events were reported in 11 (39.3%) pemphigoid cases treated with RTX. The majority of adverse events were infectious (*n* = 8). Five adverse event were severe (grade 3 of 4), and one reported death was possibly related to RTX. Noteworthy is one disease-related death in an older EBA patient, demonstrating that pemphigoid diseases can be life-threatening in therapy resistant cases and underlining the urgent need for effective treatment options. Our safety data is comparable with the reports of Maley et al. and Cho et al. who found adverse events in 33 and 31% of the MMP patients treated with RTX (18, 21). Yet, both studies observed a significantly higher adverse event rate in patients treated with conventional therapies (48 and 53%). Other studies have reported less adverse events compared to our data (23, 35–37). This might be explained by the frequent use of concomitant immunosuppressive drugs in our population with high disease severity, causing a high risk of infection.

Pemphigoid diseases appear to respond less on RTX than pemphigus, despite successful B cell depletion in the peripheral circulation in both diseases (17, 32). Furthermore, our data also showed that it takes almost 4 months (mean time till DC is 14.4 weeks) until RTX has effect, whereas in pemphigus effect is noticed within 2 months (DC at 4.0–9.3 weeks) (42–46). Possibly, B cell depletion stops pathogenic autoantibody production in both diseases, though other ongoing pathophysiological mechanisms that are not interrupted by B cell depletion might play a more substantial role in the pathogenesis of pemphigoid (27, 47). Nevertheless, PR and CR in responding pemphigoid disease patients was reached after the same time or slightly later than in pemphigus patients (32, 45, 46).

The greatest limitation of this study is its retrospective nature, which led to incomplete and/or missing data, such as objective disease scores (BPDAI and MMPDAI), and laboratory measurements of B cells and pathogenic autoantibodies at standard time points. Furthermore, a major limitation is the small sample size, especially when comparing the different pemphigoid subtypes, and the heterogeneity of our study population. The use of concomitant immunosuppressants in almost all patients (Table S1 in Supplementary Material) did not allow us to draw conclusions regarding RTX monotherapy. Nonetheless, the immunosuppressants alone did not succeed to establish DC or remission prior to RTX treatment. Moreover, the use of co-medication in severe pemphigoid diseases does reflect upon our daily practice. Lastly, it is important to emphasize that consensus late endpoints PR and CR imply to define two contrasting outcomes, but in clinical setting the difference can be minimal (one insignificant lesion once weekly vs. no lesions); therefore, patients on PR and CR might be equally satisfied with treatment result. Prospective studies with a greater sample size are needed to provide a higher level of evidence on the effectiveness of RTX in pemphigoid diseases.

In conclusion, this study demonstrated that RTX was effective in 57.1% of recalcitrant pemphigoid diseases and that the high dose regime of twice 1,000 mg was more effective than the low dose. Although relapse rates were high (66.7%), repeated RTX therapy led to remission in the majority of the relapsed cases (85.7%). An important finding is that most pemphigoid patients with IgA-dominant disease showed poor response to RTX. This finding suggests that RTX can be eliminated from the clinicians’ arsenal when encountering IgA-dominant pemphigoid patients; however, future studies are required for confirmation. RTX showed to be relatively safe. Prospective comparative studies are needed to further determine the position of RTX in the therapeutic algorithm for pemphigoid diseases.

## Author Contributions

All authors contributed to the design of the work. AL and HIE performed the data acquisition. AL performed the data analysis and all authors contributed to the interpretation of the data. Drafting the work was done by AL. All authors revised the work critically for important intellectual content and all finally approved the version to be published.

## Conflict of Interest Statement

MJ received a grant from Castle Creek and honoraria from Roche/Genentech. The authors declare that the research was conducted in the absence of any commercial or financial relationships that could be construed as a potential conflict of interest.

## Abbreviations

RTX, rituximab; BMZ, basement membrane zone; BP, bullous pemphigoid; MMP, mucous membrane pemphigoid; EBA, epidermolysis bullosa acquisita; LAD, linear IgA disease; RA, rheumatoid arthritis; DIF, direct immunofluorescence microscopy; IIF, indirect immunofluorescence microscopy; SSS, salt-split skin; DC, disease control; PR, partial remission; CR, complete remission; HIVIg, human intravenous immunoglobulin; MTX, methotrexate; AZA, azathioprine; cyclo, cyclophosphamide; MMF, mycophenolate mofetil; IV, intravenous.
