# Supplementary material for: Effectiveness and Safety of Rituximab in Recalcitrant Pemphigoid Diseases
Source: Front Immunol. 2018 Feb 19;9:248. doi: 10.3389/fimmu.2018.00248 (PMC5827539; doi:10.3389/fimmu.2018.00248)
Supplement: Supplementary file 1 [file table_1.docx]

Supplementary Material

Effectiveness and safety of rituximab in recalcitrant pemphigoid diseases

Aniek Lamberts^*^, H. Ilona Euverman, Jorrit B. Terra, Marcel F. Jonkman, Barbara Horváth

*** Correspondence:** Corresponding Author: m.a.lamberts@umcg.nl

| **Supplement 1.** Overview of all included patients with pemphigoid diseases treated with RTX | | | | | | | | | | | |
| --- | --- | --- | --- | --- | --- | --- | --- | --- | --- | --- | --- |
| **Case no/**  **Gender/ age** | **Pemphigoid subtype** | **Ig in DIF + IIF on SSS^a^** | **Medication prior to RTX** | **RTX dose first cycle** | **500mg RTX at month 6 and 12** | **Medication concomitant with RTX** | **DC** | **PR** | **CR** | **Relapse** | **Follow-up** |
| 1/f/77 | BP | IgG + IgA | prednisolone, MTX, doxycycline | 2x1000mg | yes | local steroids, prednisolone | yes | minimal therapy | no | no | Sustained PR |
| 2/m/78 | BP | IgG + IgA | prednisolone | 2x1000mg | no | local steroids, prednisolone | yes | no | no | yes | Death, not RTX related |
| 3/m/65 | BP | IgG + IgA + IgM | prednisolone, doxycycline,  dapsone, IV steroids, | 2x1000mg | yes | prednisolone | yes | minimal therapy | no | Yes | PR on retreatment with RTX |
| 4/m/72 | BP | IgG + IgA | prednisolone, MTX, doxycycline, dapsone, AZA | 2x1000mg | yes | prednisolone, azathioprine | before RTX | minimal therapy | no | no | Sustained PR |
| 5/f/56 | BP | IgG + IgA | prednisolone, MTX, doxycycline, dapsone, AZA, IV steroids, MMF, HIVIg | 2x1000mg | no | local steroid, prednisolone | yes | minimal therapy | minimal therapy | yes | Multiple times retreatment with RTX PR/CR/relapse intermittent |
| 6/m/63 | BP | IgG | prednisolone, MTX, doxycycline, dapsone, AZA, MMF, HIVIg | 2x 500mg | no | local steroid, prednisolone | yes | minimal therapy | no | yes | Multiple times retreatment with RTX: intermittent PR/CR/relapse |
| 7/f/53 | BP | IgG + IgM | prednisolone, MTX doxycycline, AZA, cyclo, MMF, HIVIg | 2x1000mg | no | one gift of HIVIg | before RTX | no | no | yes | Response on HIVIg maintenance therapy |
| 8/f/73 | BP | IgG + IgA | prednisolone, doxycycline, AZA, MMF | 2x 500mg | no | local steroid, MMF | no | no | no | n.a. | Death, possibly RTX related |
| 9/f/73 | MMP | IgG + IgA | prednisolone, doxycycline, dapsone, AZA, cyclo | 2x 500mg | no | local steroid, prednisolone, AZA | yes | no | no | yes | CR after second cycle of 2x1000mg RTX |
| 10/m/81 | MMP | IgG + IgA | prednisolone, dapsone, cyclo | 2x 500mg | no | prednisolone, AZA | no | no | no | n.a. | Lost to follow-up |
| 11/m/75 | MMP | IgG + IgA | prednisolone, MTX, dapsone, cyclo | 2x1000mg | yes | local steroid, cyclo | yes | off therapy | no | yes | CR on retreatment with RTX |
| 12/f/63 | MMP | IgG + IgA + IgM | prednisolone, dapsone, AZA, cyclo, MMF | 2x1000mg | no | local steroid | yes | off therapy | off therapy | yes | CR by local steroid |
| 13/m/62 | MMP | IgG + IgA | prednisolone, doxycycline, dapsone, cyclo, MMF | 2x1000mg | no | prednisolone, cyclo | yes | no | no | yes | DC on cyclo + prednisolone |
| 14/f/84 | MMP | IgG + IgA | prednisolone, dapsone, AZA, cyclo | 2x1000mg | no | prednisolone | yes | minimal therapy | no | no | Sustained PR |
| 15/m/46 | MMP | IgG | dapsone, cyclo | 2x1000mg | no | local steroid | yes | off therapy | no | yes | PR/CR on retreatment with RTX |
| 16/m/68 | MMP | IgG + IgA | prednisolone, cyclo | 2x1000mg | yes | prednisolone, cyclo | yes | no | no | yes | DC on prednisolone |
| 17/m/69 | MMP | IgG + IgA | prednisolone, dapsone, cylco | 2x1000mg | yes | local steroid, prednisolone, cyclo | yes | minimal therapy | off therapy | no | Sustained CR |
| 18/f/49 | MMP | IgG | prednisolone, cyclo, MMF | 2x1000mg | no | local steroid | yes | off therapy | no | yes | RTX retreatment is planned |
| 19/m/74 | MMP | IgG + IgA | prednisolone, dapsone, cyclo | 2x1000mg | no | local steroid, prednisolone, cyclo | yes | minimal therapy | no | yes | Retreatment with RTX: PR/relapse intermittent |
| 20/f/58 | MMP | IgG + IgA | prednisolone, dapsone, cyclo, MMF | 2x1000mg | no | local steroid, prednisolone | no | no | no | n.a. | No remission on kenacort injections |
| 21/f/45 | MMP | IgG + IgA | prednisolone, dapsone, cyclo. | 2x1000mg | no | prednisolone, dapsone | yes | minimal therapy | minimal therapy | no | Sustained CR |
| 22/m/62 | MMP | IgG + IgA | prednisolone, MTX, dapsone, cyclo | 2x1000mg | yes | prednisolone, dapsone | yes | minimal therapy | minimal therapy | yes | Retreatment with RTX planned |
| 23/f/59 | EBA | IgG + IgA | prednisolone, dapsone, AZA, cyclo, MMF, HIVIg | 2x 500mg | no | local steroid, prednisolone | no | no | no | n.a. | CR on prednisolone |
| 24/f/87 | EBA | IgG + IgA + IgM | prednisolone, AZA, IV steroids | 2x 500mg | no | prednisolone, AZA | no | no | no | n.a. | Death, disease related |
| 25/f/56 | EBA | IgG + IgA | prednisolone, doxycycline, dapsone, AZA | 2x1000mg | yes | local steroid, prednisolone, AZA | yes | off therapy | no | no | Sustained PR |
| 26/m/25 | EBA | **I**gG + IgA | prednisolone, doxycycline, AZA | 2x1000mg | no | local steroid, prednisolone, dapsone | yes | minimal therapy | minimal therapy | no | Sustained CR |
| 27/f/43 | EBA | IgG + IgA | prednisolone, dapsone, AZA, MMF | 2x1000mg | no | prednisolone, dapsone | no | no | no | n.a. | No remission on:  - HIVIg + dapsone + prednisolone + colchicine  - sulfasalazine + dapsone + prednisolone |
| 28/f/48 | LAD | IgA | prednisolone, dapsone | 2x1000mg | no | prednisolone | no | no | no | n.a. | No remission on:  - colchicine  - colchicine + prednisolone  - MMF  - MMF + prednisolone  - MTX  - doxycycline + niacinamide  - AZA |

RTX, rituximab; DIF, direct immunofluorescence microscopy; IIF, indirect immunofluorescence microscopy; SSS, salt split skin; DC, disease control; PR, partial remission; CR, complete remission; f, female; m, male; BP, bullous pemphigoid; MMP, mucous membrane pemphigoid; EBA, epidermolysis bullosa acquisita; LAD, linear IgA disease; MTX, methotrexate; AZA, azathioprine; cyclo, cyclophosphamide; MMF, mycophenolate mofetil; HIVIg, Human Intravenous Immunoglobulin; IV steroids, intravenous steroids; n.a., not applicable.

^a^ The dominant immunoglobuline class is underlined.
